# Preliminary indication of the role of AHL-dependent quorum sensing systems in calcium carbonate precipitation in Gram-negative bacteria

**DOI:** 10.3934/microbiol.2023035

**Published:** 2023-09-26

**Authors:** Paola Cacchio, Marika Pellegrini, Beatrice Farda, Rihab Djebaili, Silvia Tabacchioni, Maddalena Del Gallo

**Affiliations:** 1 Department of Life, Health and Environmental Sciences, University of L'Aquila, Coppito, 67100 L'Aquila, Italy; 2 Division Biotechnologies and Agroindustry, ENEA (Italian National Agency for New Technologies, Energy and Sustainable Economic Development), C.R. Casaccia, 000123 Rome, Italy

**Keywords:** bacterial CaCO_3_ precipitation, biomineralization, calcifying-bacteria, Gram-negative bacteria, quorum sensing, AHL-negative mutant, *Burkholderia ambifaria*

## Abstract

Numerous microbial species participate in precipitation of carbonates in various natural environments, including soils, geological formations, freshwater biofilms and oceans. Despite the geochemical interest of such a biomineralization process, its molecular mechanisms and adaptive aspects remain poorly known. Many Gram-negative bacteria use cell-to-cell communication systems relying on N-acylhomoserine lactone (AHLs) signal molecules to express certain phenotypic traits in a density-dependent manner, a phenomenon referred as to quorum-sensing (QS). In this work, bacterial isolates collected from cave and rhizosphere soil were analyzed to study the occurrence of the AHL-mediated QS in bacterial calcium carbonate (CaCO_3_) precipitation. To test the production of AHLs signal molecules, we cross-streaked Gram-negative calcifying strains, selected among the environmental strains studied, with the AHL-negative mutant *Chromobacterium subtsugae* strain CV026. Only *Burkholderia ambifaria* LMG 11351 was able to restore violacein production in CV026 among the tested strains. The constructed AHL-negative mutant of *B. ambifaria* LMG 11351 could not precipitate CaCO_3_ on B-4 agar. Scanning Electron Microscopy (SEM) analysis on CaCO_3_ crystals obtained *in vitro* shows crystals of different morphologies, calcified biofilms and bacteria in close contact with the precipitated crystals. In the inner layers of the bioliths deposited by *B. ambifaria* LMG 11351, a stream-like organization of the *Burkholderia* imprints was not detected by SEM. Our data provide preliminary evidence that the activation of AHL-regulated genes may be a prerequisite for *in vitro* bacterial carbonatogenesis, in some cases, confirming the specific role of bacteria as CaCO_3_ precipitating agents. We enhance the understanding of bacterial CaCO_3_ biomineralization and its potential biotechnology implications for QS-based strategies to enhance or decrease CaCO_3_ precipitation through specific bacterial processes. The AHL-negative mutant of *B. ambifaria* LMG 11351 (a well-known plant growth-promoting bacterium) could also be used to study plant-bacteria interactions. The adaptive role of bacterial CaCO_3_ biomineralization was also discussed.

## 1. Introduction

In natural environments, the chemical precipitation of calcium carbonate (CaCO_3_) is accompanied by microbial processes involving bacteria (particularly cyanobacteria), archaea, microalgae and fungi [1]–[3]. Four key factors generally govern CaCO_3_ precipitation: (i) the calcium (Ca^2+^) concentration, (ii) dissolved inorganic carbon (DIC) concentration, (iii) pH and (iv) nucleation site availability [4]. The primary role of bacteria in carbonate precipitation has been attributed to their ability to alter the chemistry of the macroenvironment through various autotrophic and heterotrophic pathways leading to an increase in pH and/or DIC concentration, eventually leading to bacterial calcium carbonate precipitation (BCP) [4]–[9]. When pH and DIC increase in an environment with abundant Ca^2+^ ions, bacterial cell walls and/or their EPS can serve as a nucleation site for the precipitation reaction [4],[10],[11]. Other mechanisms involved in BCP are the precipitation of carbonates by ion exchange across the cell membrane [5] or the effect of carbonic anhydrase (CA) [12]. Following these principles, different bacterially mediated mineralization processes have been distinguished, depending on whether they are influenced by extracellular organic molecules, induced by metabolic activity or controlled by specific genes [9]. Although many cases of biomineralization in eukaryotes involve specific genes (e.g., [13] there are presently only two documented cases of genetically controlled biomineralization in bacteria: intracellular magnetite formation by magnetotactic bacteria [14] and the intracellular amorphous calcium carbonates (iACC) biomineralization in some species of cyanobacteria [15]. Benzerara et al. (2022) identified a gene family, named *ccyA*, which proves to be a good diagnostic marker of iACC biomineralization [16]. In the environment, bacteria mediate CaCO_3_ mineralization in the form of a colony, biofilm (e.g., formation of stromatolites) or planktonic growth (i.e., free-living bacteria) [17]. Biofilms are a major mode of bacterial life in which many biomineralization processes occur in nature (e.g., [18]). A growing body of literature now suggests that CaCO_3_ minerals may be a previously overlooked component of the extracellular matrix of microbial biofilms (reviewed in [19]). It is now becoming clear that the mineral components strengthen biofilm architecture and serve as a framework to support larger bacterial populations. In addition, this mineral structure protects bacteria from antibiotics. Among emerging questions around microbial biomineralization are the cooperative formation and utilization of biominerals, which may be important to sustain microbial communities in the environment [20]. Since cells in biofilm aggregates are close, biofilms also represent an ecologically relevant context for quorum sensing (QS) [21]. For some species, there is evidence that QS is essential for constructing and dissolving biofilm communities [21]–[23].

The QS system relies on the production of a low-molecular-mass diffusible signaling molecule, the autoinducer (AI), whose extracellular concentration is related to the population density of the producing microorganism [24]–[27]. The signaling molecule can be sensed by cells and this allows them to simultaneously regulate the expression of more specific genes - to coordinate group activity - once a critical concentration corresponding to a particular cell density is reached. QS is present in Gram-negative and Gram-positive bacteria and can be used for intraspecific and interspecific communication [28]–[30]. The best characterized QS mechanism was found in Gram-negative organisms and involves AHLs as signaling molecules that differ in the structure of their N-acyl side chains [31],[32]. LuxI-type enzymes are the major producers of AHLs. The Gram-negative AIs synthesis starts from S-adenosyl-methionine (SAM) as a substrate. These molecules have an AHLs ring core and 4–18 carbon acyl chains with species-specific modifications [34]. The AIs can bind to specific cytoplasmic proteins or membrane receptors. Combined receptors function as transcription factors to regulate several genes [34]. Bacteria have evolved natural strategies to inhibit QS by producing enzymes that degrade AHLs, such as lactonases that hydrolyze the lactone ring or amide-hydrolyzing acylases AHL, releasing the homoserine lactone and the corresponding fatty acid. At the present state of knowledge, among the phenotypes regulated by QS in Gram-negative bacteria, we have luminescence, virulence, biofilm production, antibiotic production and efficiency in nodulation [33]–[35].

The ecological implications of the BCP are varied. Its ecological relevance has been exploited in biotechnology, with applications in the bioremediation of metal-contaminated soils and groundwater, soil and sand bio-consolidation, cement and CO_2_ sequestration, among others [3],[12],[35]–[39]. Although CaCO_3_ biomineralization has been studied due to its wide range of scientific and technological implications, the essential role and molecular mechanisms of this crucial geomicrobiological process are largely unknown. Some potential functions of biomineralization and its adaptive aspects were reviewed by Cosmidis and Benzerara (2022) to gain a deeper comprehension of how microbial communities function in nature and enhance our understanding of life co-evolution with its mineral environment [21]. We aimed to examine the role of QS in the calcifying process of Gram-negative (GN) bacteria. Isolates from *Alnus cordata* rhizosphere and strains belonging to our laboratory collection were tested for the *in vitro* calcification ability. To investigate the involvement of QS in the calcification process, the analysis of the production of AHLs by all the studied GN calcifying strains was performed using the biosensor *Chromobacterium subtsugae* CV026 (previously *C. violaceum*) and AHL-negative mutants of the strains that produce AHLs as signal molecules were constructed. In the present study, we found that *B. ambifaria* LMG 11351 produces AHLs and demonstrated using a quorum quenching approach that heterologous expression of the *Bacillus* sp. A24 AiiA lactonases results in loss of both AHL production and *in vitro* CaCO_3_ precipitation ability. These preliminary data indicate that QS mediated by AHLs may play a key role in the production of CaCO_3_ in Gram-negative bacteria, in some instances.

## 2. Materials and methods

### 2.1. Isolation and growth of calcifying bacteria

The heterotrophic cultivable microflora from the *Alnus cordata* rhizosphere of a garden soil near L'Aquila (Central Italy) was isolated and tested for its ability to precipitate CaCO_3_ when cultured on a Ca-rich medium. Soil subsamples (15 cm depth) were collected in a sterile container from the rhizosphere of *A. cordata* using sterile tools and stored at room temperature (less than 1 h) until microbiological analyses. Then, an amount of sample corresponding to 10 g of dried soil was resuspended in 90 mL sterile saline (0.9% NaCl) and stirred for 50 min to detach the bacteria, whose external surface is negatively charged, from soil cations [40]. Soil serial dilutions ranging from 10^−2^ to 10^−6^ were plated in triplicate on CaCO_3_ precipitation medium B-4 (10.0 g L^−1^ glucose, 4.0 g L^−1^ yeast extract, 2.5 g L^−1^ calcium acetate, 20.0 g L^−1^ agar, pH 8.0 ± 0.2) [41]. Inoculated B-4 plates were incubated at 27 °C for 7 days. All colony types were then described based on pigmentation, form, elevation, margin, opacity and surface area [42]. Colonies were classified according to morphology and chromatogenesis and characterized by cell shape and Gram type determination using the Gram 2 Kit (bioMérieux, Marcy-l'Etoile, France). Individual colonies were microscopically (Leitz-Biomed, 10X) selected based on their visual crystal formation and purified by streaking on B-4 agar medium. After uniformity and purity checks, the isolates were stored on B-4 agar slants and glycerol stocks (50% v/v, −80 °C storage).

Based on calcifying abilities, three strains (A7, A11, A14) previously isolated from a cave and *B. ambifaria* LMG 11351, from *Zea mays* rhizosphere [43], were selected among those available in the laboratory. These strains were grown on B-4 agar and, after purity checks, were further investigated together with *A. cordata* rhizosphere isolates.

### 2.2. Biolog preliminary identification

The Biolog GN2 plates (Biolog, Inc., Hayward, Calif.) were used for metabolic characterization and preliminary identification of GN calcifying bacteria. A pre-inoculum of the selected strains was prepared on Biolog Universal Growth Agar (BUG) and incubated at the optimal growth conditions of the strains. Inoculation and reading (at 590 nm) of microplates were performed according to the manufacturer's instructions using a Biolog Microstation with OmniLog. *Pseudomonas fluorescens* DSMZ 50090 was used as a reference strain. The Biolog method is based on oxidation tests of 95 substrates in a 96-well microtiter plate. Each well contains a redox dye, tetrazolium violet, allowing the colorimetric determination when microbial cells oxidize a carbon substrate. The reactions were compared to the GN database based on the similarity index, which must be at least 0.99 for acceptable species identification after 4 h of incubation and 0.75 after 16–24 h of incubation.

### 2.3. BCP assessment and SEM crystal analysis

Calcium carbonate production by bacterial isolates was evaluated by growing the strains on B-4 agar medium as described by Boquet et al. (1973) [41]. The bacterial strains were spread in triplicate on the surface of agar plates, then incubated in the presence of air at 30 °C. All plates were examined daily under a light microscope to follow crystal production for up to 7 days. A sterile medium and a medium inoculated with autoclaved bacteria were used as negative controls. SEM analysis was used to study the microstructural morphology of the *in vitro* bio-deposited crystals, microbial biofilms and their relationship with the CaCO_3_ crystals. For SEM observations, crystals were removed from the agar medium by cutting blocks and placing them in distilled boiling water for a period sufficient for the complete agar dissolution. Using this methodology, the morphology of crystals was not altered, as observed by optical microscopy both before and after their recovery [44]. The supernatant was decanted and the sediment was resuspended and washed in distilled water until the crystals were impurities-free [45]. The washed crystals were air-dried at 37°C. To observe the inner portion of the bioliths, crystals were pulverized using a mortar and pestle and observed with a Philips SEM XL30-CP.

### 2.4. Detection of AHLs by QS biosensor Chromobacterium subtsugae CV026 (autoinducer bioassay)

The AHL complementation of QS mutant *C. subtsugae* CV026 was determined by cross-streaking the GN-studied calcifying bacteria with the biosensor strain grown on Luria-Bertani (LB) agar plates (supplemented with 100 µg/mL of kanamycin). The cross-streaked plates were incubated at 30 °C for 48/72 h.

### 2.5. Construction of the AHL-negative mutant of B. ambifaria LMG 11351

In this study, a quorum-quenching approach was used to investigate the relationship between QS and *in vitro* BCP. We constructed an AHL-negative mutant of *B. ambifaria* LMG 11351 which contains the *aiiA* gene whose expression is driven by the vector's constitutive lac promoter. The constructed vector allowed the heterologous expression of the *aiiA* gene, which encodes a lactonase, from the soil *Bacillus* sp. A24, which hydrolyzes the homoserine lactone ring of the QS signals AHLs, thus rendering the QS molecules incapable of binding to the target transcriptional regulator [46]. The plasmid pME6863, a derivative of the broad-host-range vector pME6000 constructed by Reimmann et al. [47], was transferred by conjugation from *E. coli* DH5a to the wild-type *B. ambifaria* LMG 11351 strain, using *E. coli*/p2013 as a helper strain. Liquid cultures on LB medium of donor *E. coli* DH5a, helper *E. coli*/p2013, and *B. ambifaria* LMG 11351 wild-type strains were grown overnight at 30 °C with moderate shaking. Tetracycline (25 µg/mL) and kanamycin (100 µg/mL) were added to the growth medium for *E. coli* DH5a and *E. coli*/p2013, respectively. Aliquots of each bacterial culture were inoculated into tubes containing 40 mL of fresh LB medium and incubated at 30 °C for 4–5 h with shaking until an OD 600 of 1.5–2 was reached. Cells were recovered by centrifugation, washed with LB medium and resuspended in 20 mL of the same medium. Aliquots (established after OD 600 lectures) of the bacterial cultures were mixed to have 4 x 10^9^ cells of *E. coli* DH5a, 4 x 10^9^ cells of *E. coli*/p2013, and 2 x 10^8^ cells of *Burkholderia* wild-type strain. Cells collected by centrifugation were resuspended in the remaining medium and spotted onto sterile 0.45 µm filters (Millipore) on LB agar plates. After incubation for 24 h at 30 °C, the filters were washed with 1 mL of LB and 100 µL were plated on M9 minimal medium added with 3% of citric acid as the sole carbon source and 300 µg/mL tetracycline (concentration at which the wild type does not grow) [48]. Tetracycline-resistant transconjugants, identified after incubation for 48/72 h at 30 °C, were inoculated on PCAT, a semi-selective medium for *Burkholderia* containing 300 µg/mL tetracycline, for further selection [49].

## 3. Results

### 3.1. Isolation and preliminary characterization of calcifying bacteria

The list of bacteria used for this study is shown in Table 1. Most strains (B2 to B17) were new isolates from the rhizosphere of *Alnus cordata*. In addition, three bacterial isolates (A7, A11 and A14) from cave “La Grotta Nera” (Chieti, Central Italy) and *B. ambifaria* LMG 11351, from maize rhizosphere, were included. Using traditional cultivation techniques, an abundant cultivable heterotrophic microbial community was obtained from the rhizosphere of *Alnus cordata* (1.9 x 10^7^ CFUg^−1^ dry weight). It consists of bacteria (76%), actinomycetes (14%) and fungi (10%). The sixteen morphologically diverse heterotrophic colonies (B2 to B17) isolated from this microbial community were purified by streaking on B-4 agar medium and studied for their calcification ability. Microscopic observation showed that most bacterial isolates could form crystalline CaCO_3_ on B-4 agar medium (Table 1). The crystal's shape, size, position, timing and amount were established for each calcifying strain to characterize its precipitation ability *in vitro*.

**Table 1.** Precipitation activity of the studied environmental strains on B-4 agar medium at 30 °C and Gram staining results of calcifying bacteria.

Strain^a^	CaCO_3_^b^	Shape^c^	Size^c^	Position^c^	Timing^d^	Amount^e^	Gram^f^
A7	+	Spherical	Medium	c	2	Large	GN
A11	+	Spherical	Small	c/n	2	Large	GN
A14	+	Spherical/Prismatic	Large	c/n	4	Large	GN
LMG 11351	+	Spherical/Prismatic	Medium	c/n/m	2	Large	GN
B2	-	n.d.	n.d.	n.d.	n.d.	n.d.	n.d.
B3	-	n.d.	n.d.	n.d.	n.d.	n.d.	n.d.
B4	+	Prismatic/Spherical	Medium	c/n	2	Large	GN
B5	+	Prismatic/Spherical	Small	c	5	Medium	GP
B6	+	Spherical	Medium	c/n	6	Large	GN
B7	-	n.d.	n.d.	n.d.	n.d.	n.d.	n.d.
B8	+	Prismatic	Large	c	2	Large	GP
B9	-	n.d.	n.d.	n.d.	n.d.	n.d.	n.d.
B10	+	Pyramidal/Spherical	Large/small	c/n	5	Medium	GP
B11	+	Prismatic/Spherical	Medium	c	2	Small	GP
B12	-	n.d.	n.d.	n.d.	n.d.	n.d.	n.d.
B13	+	Prismatic/Spherical	Small	c/n	2	Large	GP
B14	+	Prismatic	Small	c/n	5	Medium	GP
B15	+	Spherical	Medium	c/n	2	Medium	GN
B16	-	n.d.	n.d.	n.d.	n.d.	n.d.	n.d.
B17	-	n.d.	n.d.	n.d.	n.d.	n.d.	n.d.

^a^ Strains A7, A11, and A14 were from cave, strain LMG 11351 and strains B2 to B17 were from rhizospheres; ^b^ Bacterial CaCO_3_ precipitation activity on B-4 agar at 30 °C; ^c^ Shape, size and position of the CaCO_3_ crystals biodeposited on B-4 agar medium; ^d^ Timing: number of days required to start CaCO_3_ precipitation on B-4 agar medium at 30 °C; ^e^ Amount: visually established observing each pure solid bacterial culture under light microscopy (10X); ^f^ Gram staining was also carried out on the laboratory strains A7, A11, A14, and *B. ambifaria* LMG 11351 to confirm previous results; Symbols: n.d., not determined; -, negative; +, positive for CaCO_3_ precipitation; GN, Gram-negative; GP, Gram-positive; c, on the bacterial colony; n, near the bacterial colony; m, far away in the B-4 agar medium.

**Figure 1.**
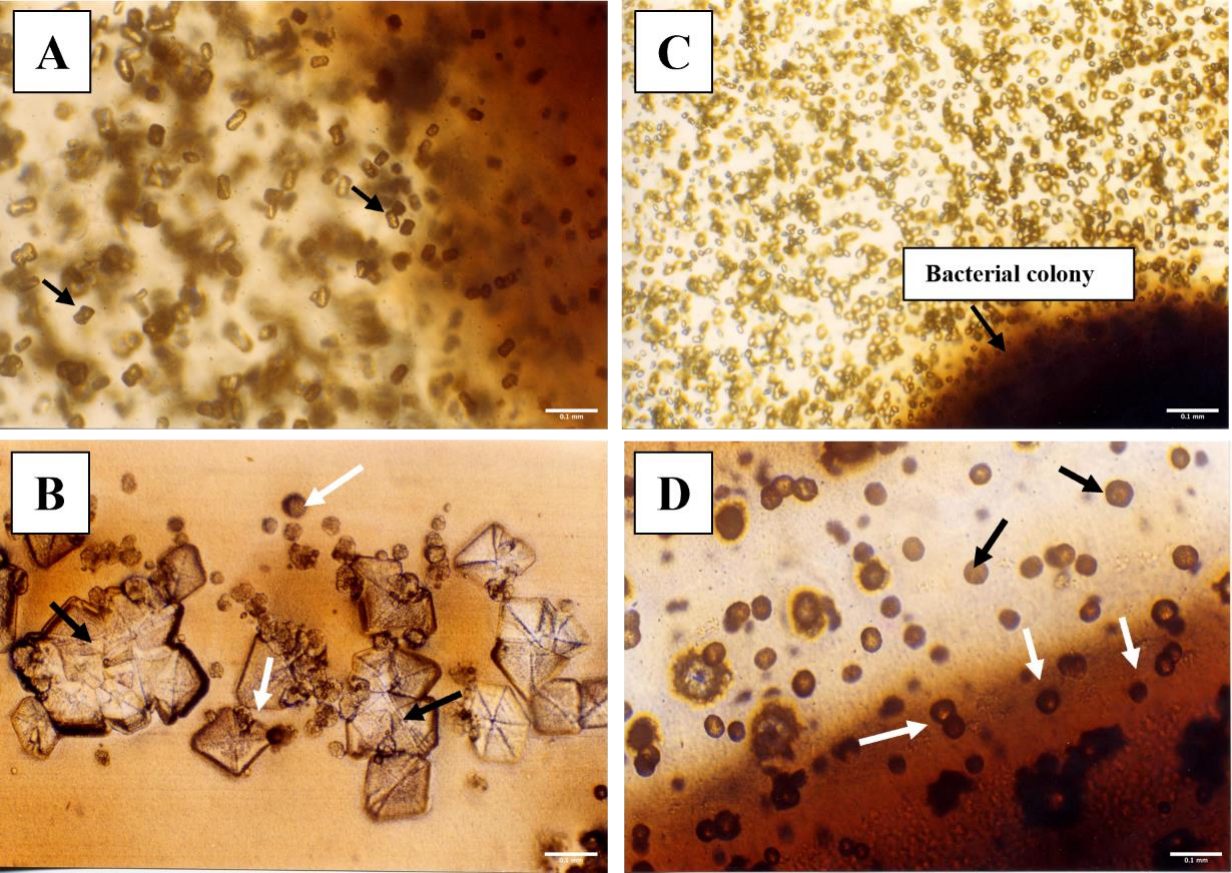
Optical images (10X) of CaCO_3_ crystals precipitated on B-4 agar medium at 30 °C by the rhizosphere isolates B4 (A), B10 (B), B13 (C) and B15 (D), after 2 days of incubation apart from isolate B10 which starts to precipitate after 5 days. Note the different sizes, morphology and position on the agar medium of the CaCO_3_ crystals deposited by the different isolates: (A) Gram-negative isolate B4 precipitated (both on and near the colony) different morphologies of crystals, including the prismatic one as shown by the black arrows; (B) Gram-positive isolate B10 deposited large pyramidal (square and hexagonal) crystals. Note the presence of crystal aggregates (black arrows) and newly deposited spherical crystals (white arrows); (C) very abundant small crystals deposited by the Gram-positive B13 isolate, both on and near the colony; (D) spherical crystals of different size deposited by the Gram-negative B15 isolate on the colony (white arrows) and in its vicinity (black arrows).

Regarding calcium carbonate precipitation activity, 9 of 16 isolates collected during this study (56%-B4, B5, B6, B8, B10, B11, B13, B14 and B15) were able to precipitate CaCO_3_ crystals. Similarly, strains A7, A11, A14 and LMG 11351 could precipitate CaCO_3_ on B-4 agar medium. The shape, size and quantity of the crystals were strain specific. Among the different crystal shapes, the prismatic and the spherical ones were the most abundant; isolate B10 precipitated pyramidal (square and hexagonal) and spherical crystals (Figure 1). No crystals were detected in the uninoculated controls or in the controls inoculated with autoclaved bacterial cells.

Table 1 also shows that 62% of the calcifying strains (A7, A11, LMG 11351, B4, B8, B11, B13, B15) started to precipitate the CaCO_3_ crystals on B-4 agar medium after 2 days of incubation at 30 °C; 23% (A14, B5, B10, B14) after 4/5 days, and only the B6 isolate started the precipitation after 6 days. All calcifying isolates precipitated crystals within the bacterial colony. Isolates A11, A14, LMG 11351, B4, B6, B10, B13, B14 and B15 precipitated CaCO_3_ crystals within and near the colony. All cave isolates (A7, A11 and A14) and 5 rhizosphere strains (LMG 11351, B4, B6, B8 and B13) showed the highest *in vitro* BCP ability. Strains LMG 11351 and B4 precipitated the most crystals in the shortest time. Moreover, strain B11 was reported to produce the lowest number of crystals.

The results of the morphological characterization of the calcifying strains collected during this study showed the prevalence (67%) of Gram-positive bacteria; only strains B4, B6 and B15 were Gram-negative. This finding is in line with the results of previous studies [41],[42]. Among all studied bacteria, 7 strains (A7, A11, A14, LMG 11351, B4, B6 and B15), all GN bacteria, were selected for further analysis based on their ability to precipitate CaCO_3_ *in vitro*.

**Table 2.** Cell morphological characterization and preliminary Biolog identification of the Gram-negative calcifying strains.

Strains^a^	Cell morphology	Identified species	% id
A7	Single rod	*Neisseria weaveri*	91
A11	Single rod	*Bordetella bronchiseptica*	99
A14	Single rod	Not identified	-
B4	Single rod	Not identified	-
B6	Single rod	*Pseudomonas syringae* pv *helianthi*	96
B15	Single rod	*Pantoea dispersa*	71

^a^ Bacterial strains A7, A11 and A14 were from cave samples; strains B4, B6 and B15 were from rhizosphere. % id = identification index (99% is the threshold of acceptability).

All selected strains were single-rod bacteria. The preliminary identification of the different isolates by means of Biolog analysis (after 24 h incubation at 30 °C) revealed that A7, A11, B6 and B15 were assigned to *Neisseria weaveri* (id 91%), *Bordetella bronchiseptica* (id 99%), *Pseudomonas syringae* pv *helianthi* (id 96%) and *Pantoea dispersa* (id 71%) species, respectively. Strains A14 and B4 were not assigned to a specific species using Biolog analysis. The reference strain *Pseudomonas fluorescens* was correctly identified (id 99%).

### 3.2. SEM analyses

**Figure 2.**
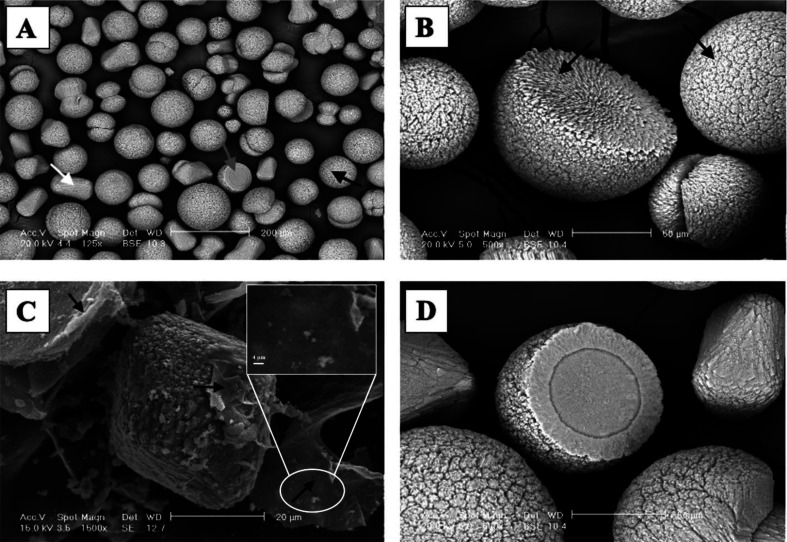
SEM images of CaCO_3_ crystals precipitated by *B. ambifaria* LMG 11351 on B-4 agar plates at 30 °C. (A) Note the presence of spherical (black arrow) and prismatic (white arrow) crystals. Spherical crystals are the most abundant. The grey arrow shows a hemispherical crystal. (B) Observe the scaly surfaces of spherical and hemispherical crystals due to the newly deposited CaCO_3_. (C) A prismatic CaCO_3_ crystal surrounded and partially covered by calcified microbial biofilms (black arrows), magnified in the inset on the top right. (D) A close-up of (A) showing a hemispherical crystal with concentric layers of crystal growth. Scale bars: (A) 200 µm; (B) 50 µm; (C) 20 µm; (D) 50 µm.

SEM was used to study crystal morphology and its relationship to microbial cells. Figure 2 shows SEM images of CaCO_3_ crystals precipitated by *B. ambifaria* LMG 11351 on B-4 agar medium at 30 °C. *B. ambifaria* LMG 11351 precipitated spherical, prismatic and hemispherical crystals (Figure 2A). The latter grew in the air-culture medium interface. Spherical crystals are the most abundant. All crystals have scaly surfaces due to the newly deposited CaCO_3_. SEM also revealed the presence of calcified microbial biofilms surrounding and partially covering the CaCO_3_ crystals (Figure 2C). In the inner layers of bioliths deposited by *B. ambifaria* LMG 11351, the typical stream-like organization of *Burkholderia* imprints observed in previous work [43] was not detected.

### 3.3. AHL production by selected GN calcifying strains and calcification activity of the AHL mutant of B. ambifaria LMG 11351

**Figure 3.**
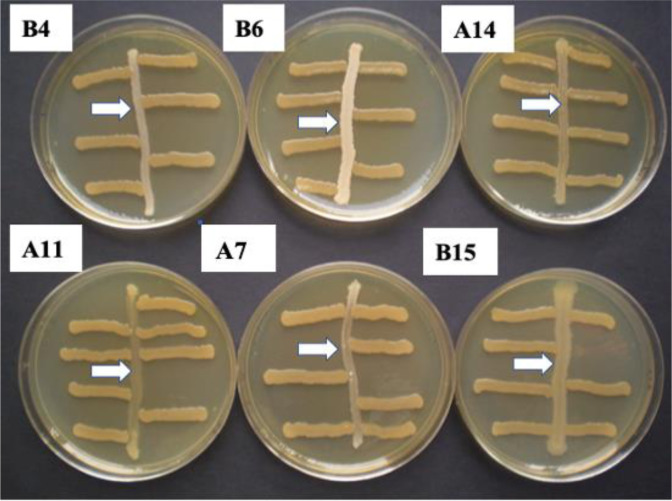
Agar plate bioassay to evaluate the induction of violacein synthesis in *C. subtsugae* CV026 by AHLs possibly produced by the selected GN calcifying isolates A7 (*Neisseria* sp.), A11 (*Bordetella bronchiseptica*), A14 (not identified), B4 (not identified), B6 (*Pseudomonas* sp.) and B15 (*Pantoea* sp.). The vertical smears of each tested bacterium (white arrows) were cross-streaked with horizontal smears of *C. subtsugae* CV026, used as a biosensor for AHLs on LB agar plates and incubated at 30 °C for 48 h. None of the isolates tested was able to induce violacein production in *C. subtsugae* CV026.

The QS-regulated violacein production in *C. subtsugae* mutant CV026 was used throughout this work for QS screening studies of the seven selected bacterial strains. In the bioassay, the GN calcifying strains were cross-streaked with the *C. subtsugae* strain CV026, unable to produce violacein. As shown in Figure 3, the calcifying GN strains A7 (*Neisseria* sp.), A11 (*Bordetella bronchiseptica*), A14 (not identified), B4 (not identified), B6 (*Pseudomonas* sp.) and B15 (*Pantoea* sp.) were unable to restore violacein production in CV026.

**Figure 4.**
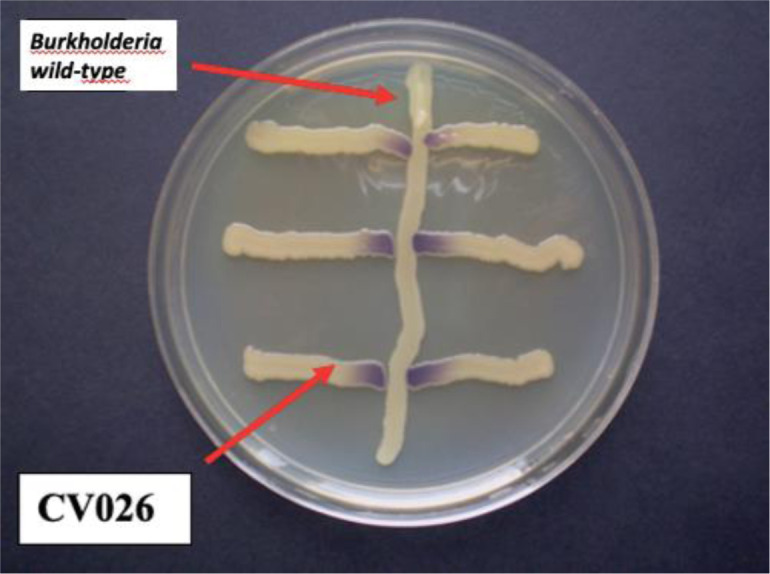
Bioassay on agar plate to evaluate the induction of violacein synthesis in *C. subtsugae* CV026 by *B. ambifaria* LMG 11351 produced AHLs. The vertical smear of the tested strain *B. ambifaria LMG 11351* was cross streaked with the horizontal smear of *C. subtsugae* CV026 (used as biosensor for AHLs) on LB agar plates and incubated at 30 °C for 48 h. Note the production of violacein by the strain *C. subtsugae* CV026 induced by AHLs produced by strain *B. ambifaria* LMG 11351.

Only the strain *B. ambifaria* LMG 11351 restored violacein production in *C*. *subtsugae* CV026 in the plate agar bioassay, as shown in Figure 4.

**Figure 5.**
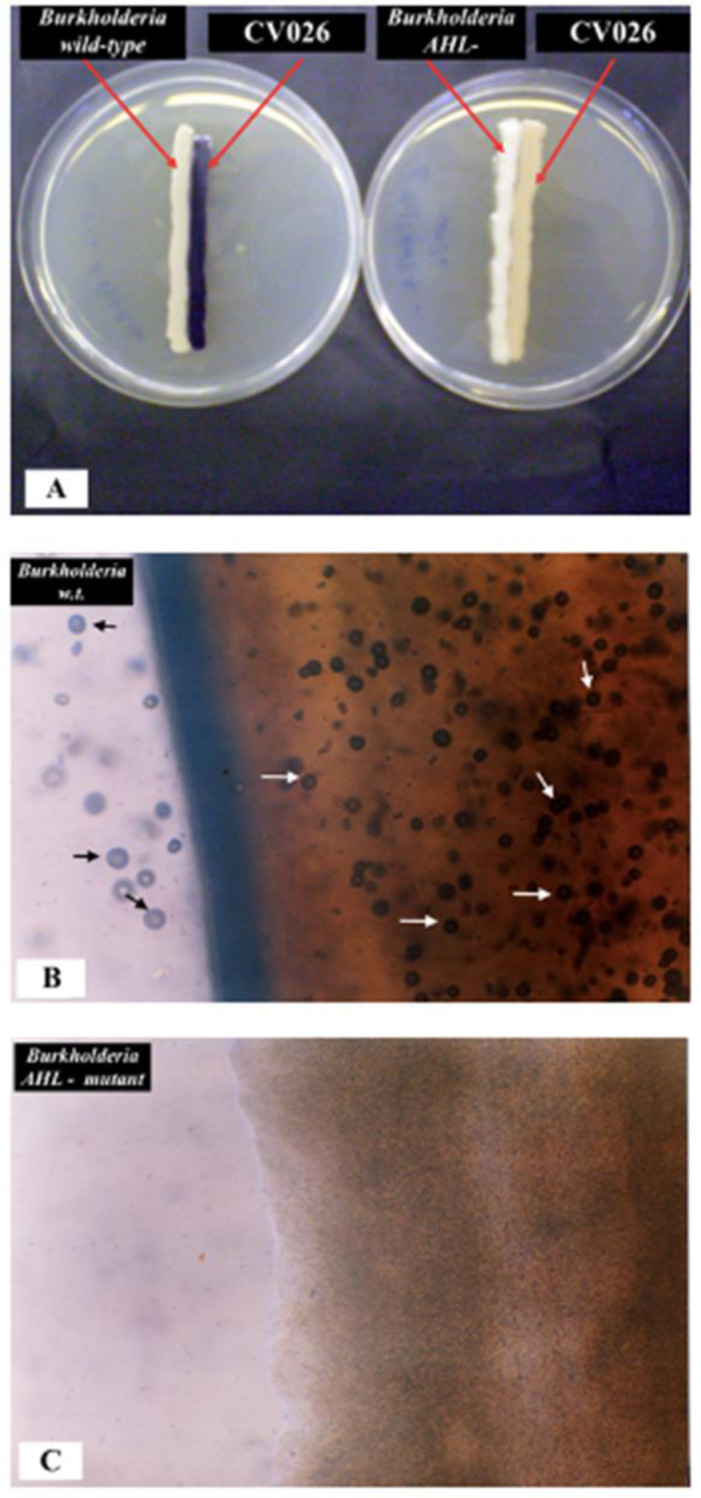
(A) Comparison bioassays on *C. subtsugae* CV026 agar plate of *B. ambifaria* LMG 11351 wild type (left) and *B. ambifaria* AHL-negative mutant (right). Production of violacein restored in *C. subtsugae* CV026 by the AHLs produced by the wild-type strain; (B) Optical image of abundant CaCO_3_ crystals biodeposited from the wild-type strain of *B. ambifaria* after 2 days incubation. Spherical and prismatic crystals were bio-precipitated on and near the bacterial colony; (C) none CaCO_3_ crystals can be microscopically observed on the colony of the *B. ambifaria* LMG 11351 AHL – negative mutant strain as well as near the bacteria, after 15 days of incubation.

As a next step, we constructed an AHL-negative mutant of *B. ambifaria* LMG 11351, which could not produce CaCO_3_ crystals on B-4 agar medium. Figure 5A shows the comparison between *C. subtsugae* CV026 bioassays of *Burkholderia* wild-type (on the left) and *Burkholderia* AHL-negative mutant strain (on the right) as well as the comparison of calcification activity observed under the microscope of the wild-type strain (B) and AHL-negative mutant (C). The AHL-negative mutant of *B. ambifaria* completely lost the ability to precipitate CaCO_3_ crystals on B-4 agar medium even after 15 days of incubation at 30 °C.

## 4. Discussion

In this study, we investigated bacterial calcium carbonate biomineralization according to both its adaptive value and its mechanism. The microscopic observations showed that in *in vitro* conditions in B-4 agar medium, 56% among the sixteen isolates from the rhizosphere of *A. cordata* and LMG 11351 from the rhizosphere of *Z. mays* were able to precipitate CaCO_3_. Strains A7, A11 and A14 from the cave shared all the capacity of crystalline formation. Metabolic activity was necessary for bacterial CaCO_3_ precipitation since no carbonate precipitation took place in the controls. The shapes, size and quantity of CaCO_3_ crystals are strain specific [50]–[52]. Results obtained in this study are consistent with previous reports [41],[45] and our previous findings [40],[50],[53] and confirm that, in appropriate conditions (especially in carbonate-rich environments such as karst cave), CaCO_3_ precipitation is present at the microbial community level, not at the individual species level (that is, that the ability to form CaCO_3_ crystals may be a so common phenotype inside a microbial community that it can be referred also to the whole community itself). On the other hand, it is known from the literature that microbial communities, particularly microbial mats, have a unique ability to alter the balance between “more reduced, and more oxidized” forms of carbon, organic matter versus CO_2_ (e.g., [54]. The present data reinforce our previous suggestion (supported by different lines of evidence based on laboratory experiments) that BCP might be relevant as an evolutionary fitness factor by maintaining the bacteria *in situ* by avoiding cell washing out from habitats most favorable for bacterial growth and survival [50],[53]. In caves, the calcifying bacteria could be selected by dripping water (which is enriched with nutrients, including calcium ions, as it crosses the layer of soil above the cave). Furthermore, in the rhizosphere, this selection could be determined by the release of organic compounds into the soil by plant roots as plant exudates [55]. Bacterial mineralization of low-molecular-weight organic acids and amino acids, naturally present in the rhizosphere environment, are important metabolic pathways involved in BCP. Our suggestion on the adaptive role of the bacterial calcification ability seems to give an additional point of view with respect to the adaptive benefits recently reviewed by Cosmidis and Benzerara (2022) (such as screening from detrimental radiation, physical protection from grazing or desiccation, resistance to antibiotics and other harmful chemical compounds, detoxification mechanisms and participation in biofilm strength and structural integrity) [21]. Microbial biofilms are of extreme clinical importance because they are associated with many persistent and chronic infections [56]. Important evidence for CaCO_3_ minerals being an intrinsic feature of the biofilm lifestyle emerged in 2015 in the Gram-negative pathogen *Pseudomonas aeruginosa* [57]. Most recently, Cynbernoknoh et al. (2022) [58] demonstrated *ex vivo* that chemical inhibition and mutations targeting CaCO_3_ precipitation significantly reduced the attachment of *P. aeruginosa* to the lung, as well as the subsequent damage inflicted by biofilms to lung tissues and restored their sensitivity to antibiotics treatment. Intriguingly, they observed the presence of bacterially produced calcite minerals in the sputum of some cystic fibrosis (CF) patients infected with *P. aeruginosa*. The GN bacteria *P. aeruginosa* and *Burkholderia cepacia* not only inhabit the same environmental niches but can also form mixed biofilms in the lungs of CF patients. In this work, the carbonate biomineralization ability of *B. ambifaria* LMG 11351 was described for the first time in the literature. To our knowledge, the first description of this phenotype in *Burkholderia* genus was made by Cacchio et al. (2012) [59] in two strains of *Burkholderia* sp. from Grave Grubbo gypsum cave (Crotone, Italy). SEM images of the interior surface of the CaCO_3_ bioliths produced *in vitro* by these old strains revealed a typical stream-like organization of the bacterial imprints, suggesting that a quorum-sensing system might be involved in the calcium precipitation process [59]. Interestingly, in the present work, unlike the other studied strains, *B. ambifaria* LMG 11351 can also promote remote (far away in the medium) carbonate precipitation in B-4 agar medium (Table 1). It suggested the involvement of specific proteins that can bind calcium ions in the mechanism of formation of carbonate crystals that could be under genetic control. It is already known in the literature the calcifying ability of members belonging to all the other genera identified in the current work, namely *Neisseria*, *Bordetella*, *Pseudomonas* and *Pantoea*. Recombinant a-carbonic anhydrase originating from *Neisseria gonorrhoeae* has been used to promote the formation of solid CaCO_3_ [60]. The investigation by Khan et al. (2021) showed the calcification ability of a *Bordetella* strain collected from rocks of Murree Hills, lower Himalayan Region, Pakistan [61]. *Pseudomonas* sp. and *Pantoea* sp. strains have been studied as candidates for bio-consolidation of ornamental stone protection by BCP [62]. Our results indicate that *B. ambifaria* LMG 11351 produces AHLs, adding this strain to the growing family of Gram-negative bacteria that produce AHLs (no scientific reports can be found about AHL production by this specific *B. ambifaria* strain). *Burkholderia cepacia* complex (Bcc) species can utilize two chemical languages: N-acyl homoserine lactones (AHLs) and cis-2-unsaturated fatty acids [63]. Furthermore, all Bcc members encode at least one QS system consisting of homologs of the *Vibrio fisheri* proteins, LuxR and LuxI, where LuxI synthesizes an AHL signal and LuxR is an AHL receptor protein that activates or represses gene expression by binding the so-called Lux-box in the promoter region of the target genes. Within the Bcc, the CepIR QS system is fully conserved. CepI directs the synthesis of C8-HSL and a minor amount of C6-HSL. Bcc species are notable for their ability to metabolize a wide range of organic compounds and to thrive in many different environments. Biofilm formation and the expression of virulence factors and secondary metabolites are controlled by quorum sensing in many Bcc strains [63]. The fact that the constructed AHL mutant is defective in calcifying in B-4 agar medium showed that activation of QS-regulated genes might be a prerequisite for *in vitro* BCP, in some instances, confirming the specific role of bacteria as CaCO_3_ precipitating agents (Figure 5). In the literature, the specific role of bacteria in BCP is mainly referred to differences in carbonic anhydrase (CA), urease expression, specific outer bacterial structures (such as glycocalyx), extracellular polymeric materials (exopolysaccharides, EPS and capsular polysaccharides, CPS) and response to calcium [51],[64],[65]. The specific role of quorum-sensing in carbonate biomineralization, established by the preliminary results of this work, may be a species/strain-specific mechanism to produce high amounts of carbonates in a short period of time consistent with the central role of CaCO_3_ scaffold in supporting biofilm morphology and other adaptive functional aspects. We provide insights into the QS-regulated functions of a well-known plant-growth-promoting rhizobacterium. Studies implicating non-pathogenic *Burkholderia* strains have demonstrated that QS is important for bacterial relationships within the rhizosphere, for interaction with plants as well as in polymicrobial communities [66],[67]. The AHL-negative mutant of *B. ambifaria* LMG 11351, constructed in the current work, may be used also to study the potential role of carbonate biomineralization in plant-bacteria interactions.

## 5. Conclusions

In this study, we focus on the maize rhizosphere isolate *B. ambifaria* LMG 11351 to investigate the role of N-acylhomoserine lactone (AHL)-dependent quorum sensing in the *in vitro* CaCO_3_ bacterial precipitation. Our results indicate that *B. ambifaria* LMG 11351 produces AHLs, adding this strain to the growing family of Gram-negative bacteria that produce AHLs (no scientific reports can be found about AHL production by this specific *B. ambifaria* strain). Moreover, we show the ability to precipitate *in vitro* CaCO_3_ crystals by *Burkholderia* species (to our knowledge, apart from the other two strains isolated in our previous work, other members of the *Burkholderia* genus have not been reported to exhibit CaCO_3_ mineralization ability). Using a quorum quenching approach, we have demonstrated that heterologous expressions of the AiiA lactonase (from *Bacillus* sp. A24) in *B. ambifaria* LMG 11351 abolish *in vitro* CaCO_3_ precipitation, suggesting an essential role of AHL-dependent QS in the control of biomineralization in this strain. Interestingly, unlike the other studied strains, *B. ambifaria* LMG 11351 can also promote remote carbonate precipitation in B-4 agar medium. We hypothesized the role of specific diffusible proteins that can bind calcium ions in the mechanism of formation of carbonate crystals that could be under genetic control. However, the underlying molecular mechanism(s) of how the QS circuitry affects CaCO_3_ precipitation in *B. ambifaria* LMG 11351 has not yet been elucidated. Our initial evidence (that AHL-dependent QS may be, in some cases, a prerequisite for *in vitro* bacterial calcium carbonate precipitation) is important and has potential scientific and biotechnology impact. This work might have technological implications for QS-based strategies to enhance (by external AHL-supplementation) or decrease (by quorum quenching) CaCO_3_ precipitation through specific bacterial processes and it can be interesting in studying plant-bacteria interactions by the AHL-negative mutant strain of *B. ambifaria* LMG 11351 (a well-known plant growth-promoting bacterium) constructed in the current work. This preliminary study also provides further insight into the significant specific role of bacteria as precipitating agents. We will investigate, as future goals, the underlying mechanism(s) of how the QS circuitry affects CaCO_3_ precipitation in *B. ambifaria* LMG 11351. Additional experiments could be performed to identify the QS-signals (e.g., HPLC-MS) as well as to assess the possible involvement of biofilm in CaCO_3_ precipitation. The main future efforts of our labs, we will aim to use a quorum-quenching approach for the screening of many Gram-negative strains to test their ability to produce CaCO_3_ in an AHL-dependent manner.
